# Good Functional Outcome after Prolonged Postanoxic Comatose Myoclonic Status Epilepticus in a Patient Who Had Undergone Bone Marrow Transplantation

**DOI:** 10.1155/2013/872127

**Published:** 2013-12-03

**Authors:** Jennifer Accardo, Domenico De Lisi, Paola Lazzerini, Alberto Primavera

**Affiliations:** ^1^Centro di Fisiopatologia del Sonno, DINOGMI, Università di Genova, Largo Rosanna Benzi, 10 16132 Genova, Italy; ^2^U.O. Anestesia e Rianimazione, DIPEA, IRCCS Azienda Ospedaliera Universitaria San Martino-IST, Largo Rosanna Benzi, 10 16132 Genova, Italy; ^3^U.O. Neurofisiopatologia, IRCCS Azienda Ospedaliera Universitaria San Martino-IST, Largo Rosanna Benzi, 10 16132 Genova, Italy

## Abstract

In anoxic coma, myoclonic status epilepticus and other nonreactive epileptiform patterns are considered as signs of poor prognosis. We report the case of a good recovery in a prolonged comatose myoclonic status epilepticus (MSE) after a cardiac arrest (CA) treated with mild therapeutic hypothermia (TH) in a patient who had undergone a bone marrow transplantation for Hodgkin's lymphoma. This case emphasizes the opportunity of performing an electroencephalogram (EEG) in the acute period after an hypoxic-ischemic insult and underlines the diagnostic difficulties between MSE and Lance-Adams syndrome, which classically occurs after the patient has regained consciousness, but can also begin while the patient is still comatose or sedated. Major problems in prognostication for postarrest comatose patients will also be pointed out.

## 1. Introduction

Postanoxic status epilepticus, particularly myoclonic status, is traditionally considered a marker of unfavorable outcome. Overall, the prognosis is extremely poor, with only a fraction of patients surviving hospital discharge and often even then reporting severe neurological or cognitive deficits [1–4].

With the advent of therapeutic hypothermia, an improvement in outcome was described in comatose survivors. In particular, some authors reported cases of patients with early post-anoxic MSE who presented a good recovery [5–7]. There are also a handful of case reports that mention early myoclonus in patients who regained consciousness and had a good neurological outcome after cardiorespiratory arrest [8–12]. These findings stress the importance of considering a combination of prognostic features before making any outcome prediction, with also bearing in mind the confounding effects of several factors, including but not limited to hypothermia and sedatives.

We describe the case of a patient who had undergone a bone marrow transplantation and had a good recovery after a prolonged post-anoxic MSE. Our case aims to demonstrate the possibility of reasonable neurological recovery despite early onset of myoclonic status even when very serious comorbidity is present. The diagnostic difficulties between MSE and Lance-Adams syndrome are also underlined.

## 2. Case Report

A 52-year-old man was diagnosed with Hodgkin's lymphoma in March 2009. He was treated with radiotherapy, five cycles of chemotherapy, and then underwent autologous bone marrow transplantation, all of which did not prove beneficial in terms of remission.

Finally, in March 2010 an allogenic transplantation was performed, obtaining a good hematologic response.

Approximately 60 days later, the patient complained of chest pain and subsequently suffered a ventricular fibrillation CA. The paramedics resuscitated him, but he remained comatose, showing no withdrawal to painful stimuli, sluggishly reactive pupils, and very weak corneal reflexes. The patient was then transported to an intensive care unit (ICU), where he was intubated and ventilated and a mild therapeutic hypothermia (target temperature 34°C) was performed over a period of 24 hours. An urgent cranial computed tomography (CT) scan was negative for hemorrhage, cerebral oedema, and structural lesions.

**Figure 1:**
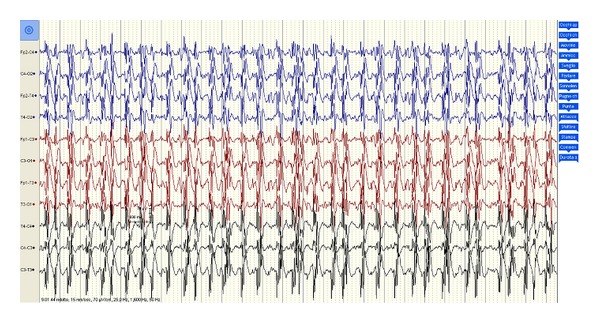
EEG pattern 36 hours after the patient suffered a cardiac arrest and treated with mild therapeutic hypothermia. After the sedative discontinuation, the EEG appeared unreactive with diffuse epileptiform discharges (polyspike-wave complexes) associated with continuous spontaneous generalized multifocal jerks, involving the face, limbs, and axial muscles.

After the sedative reduction, the patient was still comatose with a Glasgow coma scale motor (GCS-M) score <2, had no spontaneous respiration, and presented massive diffuse myoclonic jerks, related to the seizures and resistant to phenytoin, diazepam midazolam, and propofol. An EEG was performed 36 hours after insult, showing a pattern suggestive of unreactive MSE (Figure 1). Levetiracetam was added at the dose of 2000 mg/die and later increased to 3000 mg/die, without apparent improvement.

**Figure 2:**
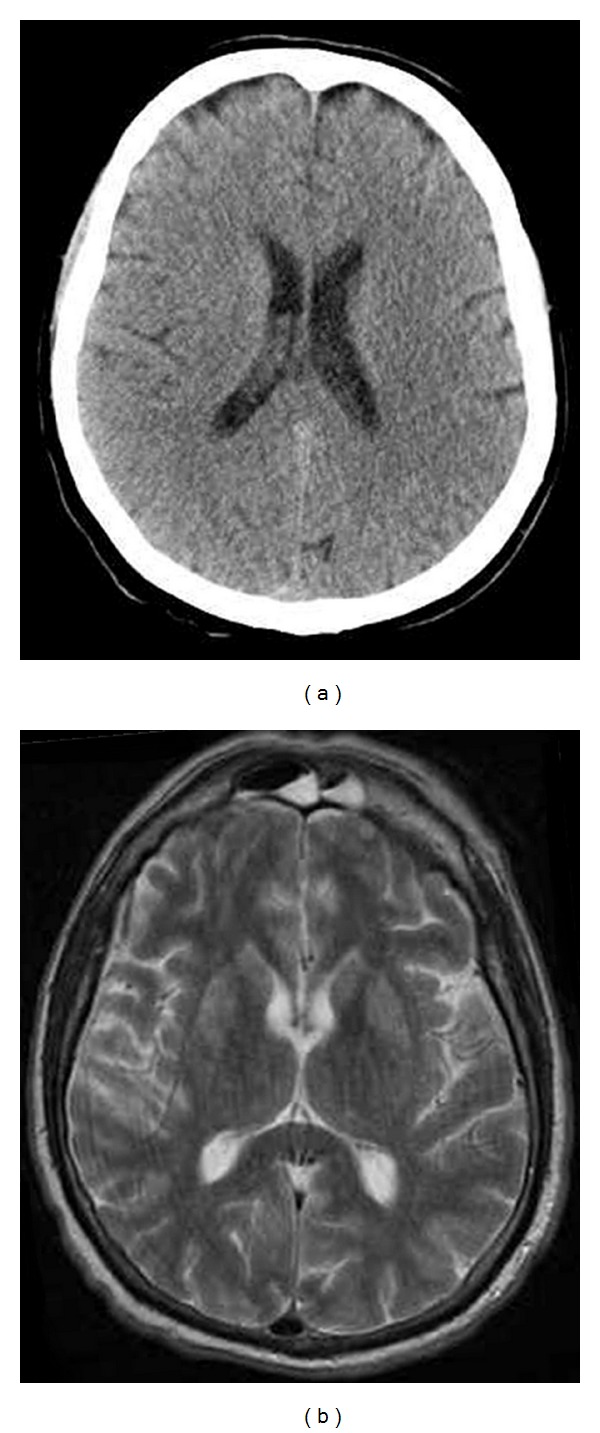
(a) An urgent cranial computed tomography scan was negative for cerebral oedema, hemorrhage, and structural lesions. (b) The magnetic resonance imaging, obtained three weeks after the initial event, demonstrated a mild hyperintensity in the lenticular nuclei and periventricular areas bilaterally in T2 sequences.

Magnetic resonance imaging (MRI), obtained three weeks later, demonstrated a mild hyperintensity in the lenticular nuclei and periventricular areas bilaterally in FLAIR and T2 sequences (Figure 2).

**Figure 3:**
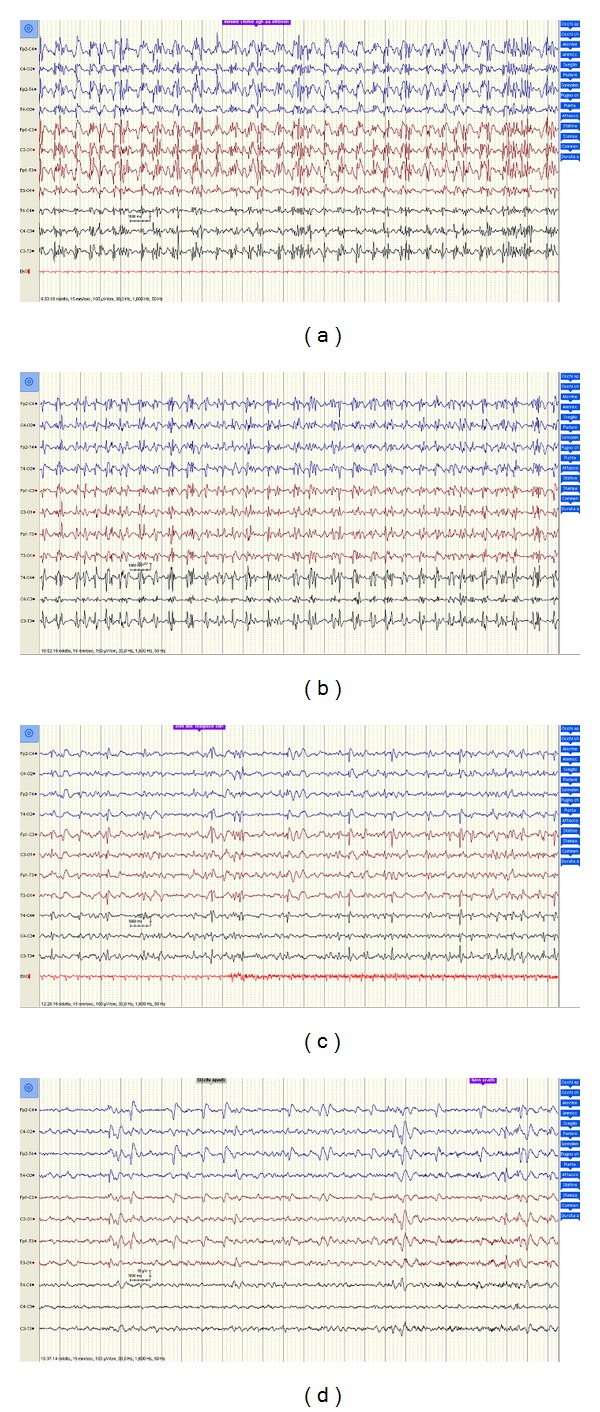
Repeated EEG recordings obtained (a) 4 days, (b) one week, (c) two weeks, and (d) one month after the initial insult confirmed the presence of anomalies, including polyspike-waves, sometimes followed by brief depressions of the electrical activity, even though a tendency toward a gradual quantitative decrease of pathologic potentials was noted.

Repeated EEG recordings confirmed the presence of anomalies, including recurrent polyspike waves, sometimes followed by brief depressions of the electrical activity (Figure 3), even though a tendency toward a gradual quantitative decrease of irritative potentials was noted. Diffuse, generalized, unrelenting myoclonic jerks involving the face, trunk, and limbs persisted and sometimes appeared to be stimulus related.

Due to the patient's severe general conditions, immunosuppressive therapy was suspended. On day 10 after the initial event, a tracheostomy was performed. Eye opening occurred on day 14, while at day 36 the patient occasionally presented with brief deviation of eyes towards the source of stimulation. In the next days, the patient slowly started to regain consciousness and was able to interact with the surrounding environment and feed autonomously, while until that moment a parenteral nutrition was needed. Myoclonic jerks persisted, but they became less frequent. The patient was discharged on day 80, after a cycle of rehabilitation to help him reacquire the ability to ambulate.

**Figure 4:**
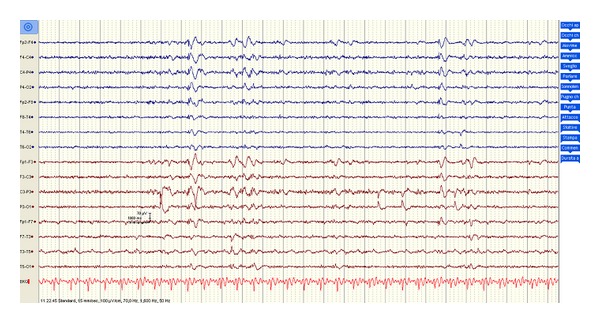
A follow-up EEG obtained seven months later revealed a normal background activity, although isolated diffuse polyspike discharges time-locked with myoclonic jerks persisted, more evidently in the anterior regions.

During the following months, a mild cognitive recovery was achieved (MMSE: 20/30), even though he presented with acalculia, agraphia, dysarthria, and action myoclonus, especially in relation with movement and emotional stimuli. A follow-up EEG performed seven months after the initial insult demonstrated a normal background activity, although isolated brief polyspike discharges appeared, in association with the myoclonic jerks (Figure 4). The patient also underwent a follow-up cerebral MRI with Turbo-FLAIR and DWI sequences, which did not reveal the previously reported hyperintensity involving the lenticular nuclei, while it showed a reduction of the hyperintensity involving the lateral periventricular areas bilaterally.

At present (three years after the initial event), the patient has an improved cognitive performance with an MMSE score of 23/30 and is sufficiently autonomous with a Glasgow-Pittsburgh cerebral performance category scale score of 2, while the intensity of myoclonus has reduced, although Lance-Adams syndrome persists. His therapy consists of low doses of Diazepam, Levetiracetam 1000 mg two times a day, and speech therapy. He still undergoes hematologic and neurologic follow-up visits on a three months basis.

## 3. Discussion

The prognosis of a patient after a CA is influenced by multiple factors, including age, presence of comorbidities, circumstances and duration of arrest, characteristics of resuscitation and cardiac rhythm, duration of impaired consciousness after the event, and presence of seizures.

In the case we presented, the patient had multiple factors suggesting an unfavorable outcome.

The presence of serious comorbidities, which consisted in Hodgkin's lymphoma requiring two bone marrow transplantations, could be considered an important element indicating a poor prognosis.

A prolonged coma following nontraumatic cerebral injuries also carries a very poor prognosis. In postcardiac arrest patients treated with therapeutic hypothermia, time to awakening after resuscitation is highly variable and often no longer than three days. Earlier awakening is associated with better neurologic status at hospital discharge. In our patient's case, the impairment of mental status lasted for more than a month.

Another indicator of severe prognosis in this patient was the presence of myoclonus after sedative medications were reduced, in association with an unreactive EEG pattern consistent with posthypoxic status epilepticus (SE) lasting at least 20 days. The EEG classification and its variations over time have been shown to hold a prognostic value for favorable and unfavorable outcomes. “Malignant” EEG patterns include myoclonus with EEG correlate, a nonreactive background, burst suppression, and SE; nevertheless, sedation during hypothermia might create an iatrogenic, reversible burst-suppression pattern and preclude reactivity in some patients.

In adult patients with SE, age, history of prior seizures or epilepsy, SE aetiology, level of consciousness, seizure type at SE onset, seizure duration, need for mechanical ventilation, and development of acute complications are the major prognostic determinants [13, 14]. Only in the last few years the electroclinical differences between proper and comatose forms of SE have been emphasized [15, 16].

Postanoxic SE, in particular, is a strong and independent predictor, associated with approximately 100% mortality rates. Misdiagnosis of nonconvulsive status epilepticus (NCSE) as severe anoxic damage can falsely suggest a poor prognosis [17]. For this reason, patients with hypoxic-ischaemic encephalopathy were frequently excluded from data series [18].

It should be outlined that controversy as to the EEG definition of seizures and SE still exists, which, together with a high variability in the timing of imaging and EEG, is likely to contribute to the interinstitutional variations seen in seizure rates in CA treated with TH series.

Rossetti and Logroscino [19] emphasized that postanoxic catastrophic myoclonus is generally transient, lasting 24–48 hours, while myoclonic Lance-Adams syndrome is compatible with restoration of consciousness. Although several studies, including those using continuous EEG (c-EEG) recordings, found 100% mortality in patients with myoclonus [20], there are CA patients treated with hypothermia who developed myoclonus and yet had good outcomes. These patients constitute a small but important minority [5–7]. It is possible that hypothermia has an ameliorating effect, not only on the hypoxic-ischemic insult but also on the damaging effect of seizures.

The good recovery described in some cases and the overlapping with Lance-Adams syndrome emphasize the opportunity of a subcategorization based, at least, on etiology, duration of the altered state of consciousness, neuroimaging features, and clinical and neurophysiological findings.

Notwithstanding the presence of factors indicating a severe prognosis, some other elements were present in our patient that could indicate a more favorable outcome, such as the arrest being due to a ventricular fibrillation as opposed to pulseless electrical activity or asystole.

Data derived from neuroimaging also showed no oedema or structural lesions in the acute phase, which could arguably indicate that the cerebral suffering and damage were limited and the patient's compromised consciousness could have partially been a result of SE rather than anatomic injury. Preliminary studies indicate that quantitative MRI has strong predictive value after CA and TH, since it can predict the long-term functional and cognitive impact of hypoxic-ischemic encephalopathy, especially when performed within five days after the event [21, 22].

Other factors indicated the possibility of a favorable outcome: more specifically, the sluggish pupil reactivity and weak corneal reflex suggested some preservation of brain stem functions.

In this case, it could be useful to consider that posthypoxic myoclonus is generally divided into two entities, acute and chronic posthypoxic myoclonus.

Acute posthypoxic myoclonus, also known as myoclonic status epilepticus (MSE), typically begins within 24 hours from the hypoxic insult and occurs in patients who are deeply comatose, even after the discontinuation of sedative drugs. It consists of continuous (usually massive) myoclonus, with rhythmic or irregular bilateral synchronous jerking of face, trunk, and limbs, often with repetitive blinking, eye opening, upward eye rolling, and mouth twitching. The EEG shows generalized, bisynchronous polyspikes, spikes or sharp waves preceding and time-locked with the clinical myoclonus, superimposed on a diffusely slow and suppressed background or burst-suppression pattern. The treatment of this condition is difficult and of questionable usefulness, since it is associated with poor prognosis. Nevertheless, prolonged seizures may cause cerebral injury and should be treated promptly and effectively with benzodiazepines, sodium valproate, propofol, levetiracetam, or a barbiturate.

Chronic posthypoxic myoclonus typically occurs within a few days to a few weeks after the hypoxic injury. First described by Lance and Adams in 1963, it is a multifocal action myoclonus in combination with startle-sensitive, bilateral, and generalized jerks and is usually accompanied by dysmetria, dysarthria, and ataxia, with relative preservation of higher cognitive functions. It classically occurs after the patient has regained mental status but can begin while the patient is still comatose or sedated [8, 9, 12]. The prognosis is generally favorable and these patients continue to improve over time although cerebellar signs may persist. The EEG shows a cortical origin with responsive cortical rhythms which progressively regain normal patterns. Chronic posthypoxic myoclonus has been shown to respond especially to valproate, levetiracetam, and clonazepam.

It is vital to accurately distinguish between MSE and Lance-Adams syndrome, due to their very different prognoses. Although the distinction between these two conditions may be favored by the aforementioned aspects, a correct diagnosis is sometimes complicated, since there is a considerable clinical overlap and several confounders are usually present.

These considerations lead us to think that it was appropriate to describe our patient as initially suffering from MSE, which later evolved to fulfill the criteria for Lance-Adams syndrome that Yadavmali and Lane [11] suggested. However, the majority of authors prefer to consider this evolution as an initial misdiagnosis of Lance-Adams syndrome [9, 23].

In summary, when evaluating comatose postarrest patients with myoclonus, it should be borne in mind that clinical assessment is often unreliable. Ancillary investigations can add valuable information, although they have traditionally been underused and there is no established algorithm of clinical signs or investigations which allow a definitive prognosis. Blondin and Greer [24] suggested that the American Academy of Neurology practice parameters for assessing prognosis after CA may not be accurate for patients treated with TH and may lead to an overly pessimistic prognostication and premature withdrawal of care [9, 23].

## 4. Conclusion

The patient described here developed a post-anoxic MSE and was unconscious for a prolonged time span, at least longer than 35 days, however, in absence of remarkable neuroimaging signs of cerebral anoxia. Recovery of consciousness was gradual, but a Lance-Adams syndrome persisted at followup, after 3 years. We suggest that MSE was the early manifestation of an hypoxic-ischemic encephalopathy and later evolved to fulfill the criteria for Lance-Adams syndrome.

It is mandatory to perform a global clinical, neurophysiological, and radiological evaluation of the patient before establishing a prognosis, since an inaccurate prediction of poor outcome may result in the patient being denied potentially life-saving treatments. As Benson and Young [25] suggested, myoclonus may not be always the “kiss of death.”
